# Viral Filtration Efficiency of Fabric Masks Compared with Surgical and N95 Masks

**DOI:** 10.3390/pathogens9090762

**Published:** 2020-09-17

**Authors:** Harriet Whiley, Thilini Piushani Keerthirathne, Muhammad Atif Nisar, Mae A. F. White, Kirstin E. Ross

**Affiliations:** Environmental Health, College of Science and Engineering, Flinders University, GPO Box 2100, Adelaide 5001, Australia; Thilini.Ke@flinders.edu.au (T.P.K.); Muhammadatif.Nisar@flinders.edu.au (M.A.N.); Mae.White@flinders.edu.au (M.A.F.W.); Kirstin.Ross@flinders.edu.au (K.E.R.)

**Keywords:** cloth mask, COVID-19, public health, PPE, face mask

## Abstract

In response to the Coronavirus Disease 2019 (COVID-19) pandemic, current modeling supports the use of masks in community settings to reduce the transmission of SARS-CoV-2. However, concerns have been raised regarding the global shortage of medical grade masks and the limited evidence on the efficacy of fabric masks. This study used a standard mask testing method (ASTM F2101-14) and a model virus (bacteriophage MS2) to test the viral filtration efficiency (VFE) of fabric masks compared with commercially available disposable, surgical, and N95 masks. Five different types of fabric masks were purchased from the ecommerce website Etsy to represent a range of different fabric mask designs and materials currently available. One mask included a pocket for a filter; which was tested without a filter, with a dried baby wipe, and a section of a vacuum cleaner bag. A sixth fabric mask was also made according to the Victorian Department of Health and Human Services (DHHS) guidelines (Australia). Three masks of each type were tested. This study found that all the fabric masks had a VFE of at least 50% when tested against aerosols with an average size of 6.0 µm (VFE_(6.0 µm)_). The minimum VFE of fabric masks improved (to 63%) when the larger aerosols were excluded to give and average aerosol size of 2.6 µm (VFE_(2.6 µm)_), which better represents inhaled aerosols that can reach the lower respiratory system. The best performing fabric masks were the cotton mask with a section of vacuum cleaner bag (VFE_(6.0 µm)_ = 99.5%, VFE_(2.6 µm)_ = 98.8%) or a dried baby wipe (VFE_(6.0 µm)_ = 98.5%, VFE_(2.6 µm)_ = 97.6%) in the pocket designed for a disposable filter, the mask made using the Victorian DHHS design (VFE_(6.0 µm)_ = 98.6%, VFE_(2.6 µm)_ =99.1%) and one made from a layer of 100% hemp, a layer of poly membrane, and a layer of cheesecloth (VFE_(6.0 µm)_ = 93.6%, VFE_(2.6 µm)_ = 89.0%). The VFE of two surgical masks (VFE_(6.0 µm)_ = 99.9% and 99.6%, VFE_(2.6 µm)_ = 99.5% and 98.5%) and a N95 masks (VFE_(6.0 µm)_ = 99.9%, VFE_(2.6 µm)_ = 99.3%) were comparable to their advertised bacterial filtration efficacy. This research supports the use of fabric masks in the community to prevent the spread of SARS-CoV-2; however, future research is needed to explore the optimum design in ensuring proper fit. There is also a need for mass education campaigns to disseminate this information, along with guidelines around the proper usage and washing of fabric masks.

## 1. Introduction

In response to the global Coronavirus Disease 2019 COVID-19 pandemic caused by SARS-CoV-2, there has been increasing support for the wearing of masks in community settings [1,2,3,4,5]. On 15 April 2020, the US CDC recommended the use of cloth face covering, especially in areas of significant community based transmission [4]. This was followed by the World Health Organization recommendation, on the 5 June, that masks can be used in community settings to protect oneself when in contact with an infected individual or for source control (worn by healthy and potentially asymptomatic individuals to prevent onward transmission) [6]. On the 22 July in Australia, the second wave of SARS-CoV-2 cases in Victoria led to mandatory wearing of masks in metropolitan Melbourne and Mitchell Shire, which was enforced by the police through the issuing of fines [7]. This was quickly followed by companies across Australia recommending the use of masks for workers and customers [8].

Modeling studies support the use of masks in the community to prevent the spread of COVID-19 [3,9]. Eikenberry et al. [3] used a hypothetical mask adoption model to demonstrate that if 80% of the community in New York wore masks in public, and the masks were 50% effective, this could prevent 17–45% of projected number of deaths. The same study found that even masks that are less effective could significantly reduce the number of deaths in areas with low transmission rates. For example, in Washington if 80% of the community wore masks that were only 20% effective this could still reduce the number of deaths by 24–65%.

Despite the evidence from modeling studies that support guidelines for mask wearing in the community, this advice has received some backlash [10]. One of the main arguments against the use of face masks in community settings is the limited availability of medical masks and the need to triage supplies and ensure healthcare workers have adequate protection [4,11]. For example, on Twitter at the beginning of the pandemic, the US Surgeon General urged people against buying masks for use by healthy people [9]. The need to triage the use of medical supplies has led to the emerging support for the use of fabric face masks [4,6]. However, there is currently limited evidence available regarding the efficacy of fabric face masks to prevent respiratory infections [12].

This study used a standard method to evaluate the efficacy of currently available fabric face masks to filter a model virus compared with surgical and N95 masks. This information will inform best practice for fabric face mask design to protect against respiratory diseases and reduce community-based transmission of SARS-CoV-2.

## 2. Results

The viral filtration efficiency (VFE) of the masks tested in this study is presented in Table 1. All the fabric masks had a VFE of at least 50% against aerosols with an average size of 6.0 µm (VFE_(6.0 µm)_) and this improved to 63% against aerosols with an average size of 2.6 µm (VFE_(2.6 µm)_), which represents the size of aerosols that can reach the lower respiratory system. The best performing of the fabric masks was the cotton fabric mask with a pocket that allowed a section of vacuum cleaner bag (VFE_(6.0 µm)_ = 99.5%, VFE_(2.6 µm)_ = 98.8%) or a dried baby wipe (VFE_(6.0 µm)_ = 98.5%, VFE_(2.6 µm)_ = 97.6%) to be inserted. Similarly effective, was the mask made from two layers of reusable shopping bag (nonwoven polypropylene) and one layer of cotton (according to Victorian DHHS guidelines) (VFE_(6.0 µm)_ = 98.6%, VFE_(2.6 µm)_ =99.1%), followed by the mask made with an outer layer of 100% hemp, a middle layer of poly membrane, and an organic cheesecloth inner (VFE_(6.0 µm)_ = 93.6%, VFE_(2.6 µm)_ = 89.0%). The VFE of the N95 (VFE_(6.0 µm)_ = 99.9%, VFE_(2.6 µm)_ = 99.3%) and surgical masks (VFE_(6.0 µm)_ = 99.9% and 99.6%, VFE_(2.6 µm)_ = 99.5% and 98.5%) were comparable to the bacterial filtration efficiency reported on their packaging.

## 3. Discussion

Current recommendations regarding the wearing of fabric masks to reduce the spread of SARS-CoV-2 has elicited much debate [10]. Concerns have been raised regarding the lack of evidence on the efficacy of fabric masks and the potential risks, such as a false sense of security which may lead to a disregard of social distancing measures, contamination through adjusting and touching with contaminated hands, and improper fit [10,14,15].

This study showed that fabric masks currently available for purchase had a minimum viral filtration efficiency of 50%. This was significantly enhanced through the use of a section of a vacuum cleaner bag or a baby wipe as a substitute for a pocket filter. There were also two designs with three layers of different fabrics (Fabric 6 and Fabric 3) which performed exceptionally well with VFE above 90%. This finding supports the recommendations from The World Health Organization on making your own fabric masks [6].

The results from this study are supported by other studies that have assessed the ability of fabric masks to filter particles. A study on the filtration efficiency of various fabrics found that the removal efficiency when one layer of fabric was used range from 5% to 80% and 5% to 95% for particle sizes of <300 and >300 nm, respectively. However, this was significantly improved when multiple layers of different combinations of fabric were used. For example cotton–silk, cotton–chiffon, cotton–flannel fabric combination filtered more than 80% of particles <300 nm and >90% of particles >300 nm [16]. Another study conducted in Taiwan recruited volunteers with confirmed influenza A and B and suspected COVID-19 and asked them to wear a medical mask or a three-layer cotton mask in a bedroom or a car. The authors then measured the particles (with a size range of 20–1000 nm) located within 1 m of the individual for 1 h and found no significant difference in the particles produced from coughing or sneezing between the participants wearing cotton masks and those wearing medical masks.

One limitation of this study is that it does not take into consideration the fit of the mask. Future research is needed to examine this issue to inform the design and fit of fabric, as Konda et al. [16] demonstrated that gaps due to improper fit of a fabric mask can result in over a 60% decrease in the filtration efficiency. Another limitation is that the standard method used in this study challenges masks with the viruses traveling at the flow velocity associated with breathing. Coughing and sneezing result in faster flow velocities which could affect the viral filtration efficiency [17].

There is also the need for education campaigns aimed at informing individuals on how to wear fabric masks. This should include details on the best design and importance of good fit. There should also be advice on proper usage, including how to don and doff face masks, the importance of not touching masks to prevent self-contamination, and the need to wash masks in >60 °C water with soap or laundry detergent [6]. However, given the success of current handwashing and social distancing campaigns, mass education on the face usage of mask is possible [1].

## 4. Materials and Methods

### 4.1. Face Masks

**Figure 1:**
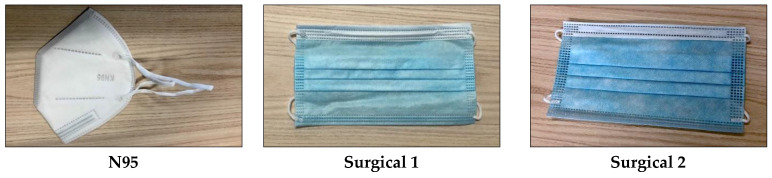

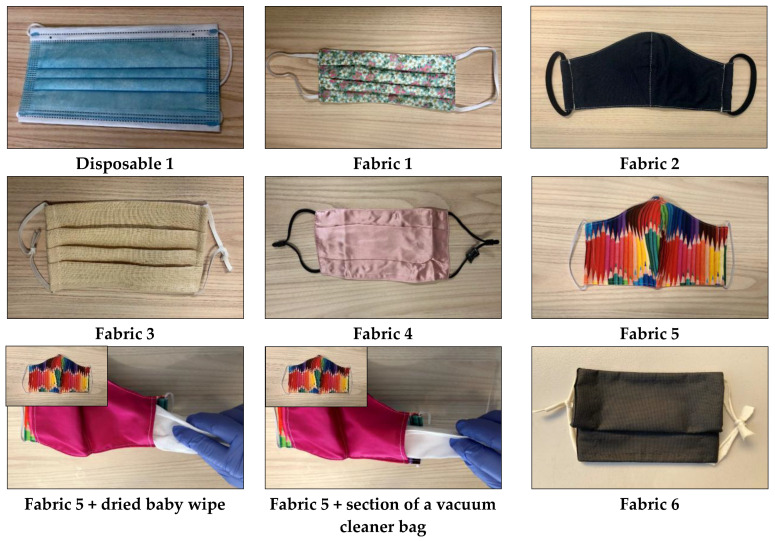
Masks tested in this study.

Fabric face masks were purchased from five Etsy retailers (www.etsy.com.au) based in Australia and chosen at random. Five different types of fabric face masks were selected to best represent the most common types of fabric masks currently available for purchase. One of the selected face masks was designed with a pocket for a filter; however, given that there are limited filters available a dried baby wipe and a section of a vacuum cleaner bag were tested instead of a mask filter. A final fabric face mask was also made in accordance with the design provided by the Victorian Department of Health and Human Services [13]. For comparison with the fabric face masks, two different types of surgical masks, a disposable face mask and an N95 mask were also purchased in Australia. The masks tested in this study and shown in Figure 1 and descriptions are included in Table 1. Three of each of the different types of masks were tested.

### 4.2. Conditioning of Face Masks Prior to Testing

Each mask was conditioned for a minimum of 4 h at a temperature of 21 ± 5 °C and relative humidity of 85% ± 10% prior to testing.

### 4.3. Bacteriophage MS2 Preparation

Bacteriophage MS2 (ATCC 15597-B1) was propagated using the double agar layer method. The bottom layer (of the tryptone soya agar (TSA) *Escherichia* *coli* agar plates) consisted of TSA (Oxoid, Basingstoke, Hampshire, UK) and the top layer consisted of 4.5 mL of soft TSA mixed with 500 µL of overnight *E. coli* (ATCC 700891) culture (which had been incubated overnight at 37 °C in typtone soya broth (Oxoid)) and 200 µL of freeze thawed MS2 bacteriophage solution. The plates were then incubated overnight at 37 °C. The plaques were harvested in peptone water (Oxoid) and purified by centrifugation at 3000 rpm for 15 min to separate the host cell debris and the bacteriophage. The supernatant was filtered through a 0.22 μm Millex-GP Syringe Filter Unit (Millipore, catalog number SLGP033RS, Tullagreen, Cork Ireland) and used as a stock solution. This stock was serially diluted in sterile water and the concentration was determined by plating and counting plaques using the double agar layer method described above.

### 4.4. Viral Filtration Efficiency

Mask testing was carried out in accordance with the ASTM F2101-14 Standard Test Method for Evaluating the Bacterial Filtration Efficiency (BFE) of Medical Face Mask Materials, Using a Biological Aerosol of *Staphylococcus aureus* [18]. However, the method was modified, and *S. aureus* was replaced with bacteriophage MS2 as the test specimen. This modification was made as *S. aureus* has a diameter of ≈1 μm [19], which is roughly 12 times larger than the SARS-CoV-2 virion (70–90 nm in diameter [20]). Given, the public health significance of these findings, the precautionary principle was applied to the experimental design and MS2 (diameter of 27 nm) was chosen as the model microorganism as it is 2–3 times smaller than SAR-CoV-2 [21].

**Figure 2:**
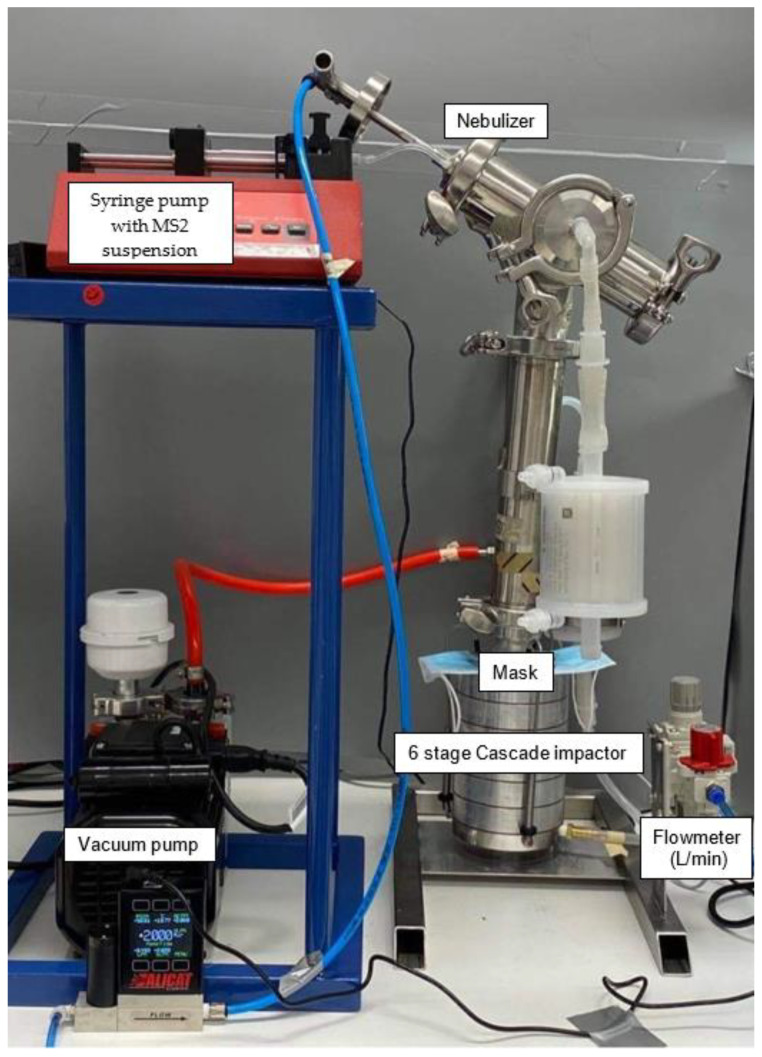
Mask testing rig, set up according to the ASTM F2101-14.

Briefly, masks were challenged (see Figure 2 for challenge apparatus) with 200 µL of 8.3 × 10^5^ PFU/mL MS2 viral aerosols in sterile water at a flow rate of 28.3 L/min, which is within the range of normal respiration and the limitations of the cascade impactor [18]. Masks were placed facing out to test their filter efficacy when used as a personal protection device. The pressure was maintained at 35 kPa and the challenge suspension was delivered for 1 min.

Virus aerosols that passed through the mask were captured on TSA-*E. coli* plates within the six-stage cascade impactor. These plates were then incubated overnight at 37 °C. The plaques were counted and recorded as positive hole corrected [22]. The positive hole corrected counts for each of the six stages were added together and the total from the three trials was averaged. Positive control runs were performed in triplicate without a mask clamped into the test system to determine the number of viable MS2 aerosols being generated. Negative control runs were performed in triplicate by collecting a 2 min samples of air from the aerosol chamber without the MS2. The average aerosol size was 6.0 µm, which is within the size range of aerosols produced by coughing (0.62–15.9 μm) [23]. Viral filtration efficiency (VFE) was calculated by comparing the average positive hole corrected PFU of MS2 captured after the mask compared with the positive control. The VFE for each mask was also calculated with the larger aerosol removed to provide an average aerosol size of 2.6 µm, which better represents the size of inhaled aerosol that reaches the lower respiratory system and alveolar region (<3 µm) [24].

## 5. Conclusions

This study demonstrated that typically available fabric masks have at least a 50% viral filtration efficiency and this can be increased through the use of everyday items (vacuum cleaner bag and baby wipes) as an alternative to a disposable pocket filter or through designing fabric masks to have three layers of different fabrics. This research supports the use of fabric masks in community settings to prevent the spread of SARS-CoV-2. Future research is needed to investigate fabric mask designs that allow the best fit, examine the influence of different flow velocities, and determine the availability and costs of materials needed to make efficient masks. Additional substitutes to filters should be tested to ensure there is global access to the supplies needed to produce effective masks and reduce the spread of SAR-CoV-2.

## Figures and Tables

**Table 1:** Average viral filtration efficiency (VFE) of different types of fabric masks compared with N95, surgical, and disposable masks determined using ASTM F2101-14 standard method with bacteriophage MS2 as the challenge virus.

Mask	Average Viral Filtration Efficiency for an Average Aerosol Size of 6.0 µm (VFE_(6.0 µm)_) (%) [Range]	Average Viral Filtration Efficiency Calculated with the Larger Aerosols Excluded to Give an Average Aerosol Size of 2.6 µm (VFE_(2.6 µm)_) (%) [Range]	Description ^1^
N95	99.9 [99.8–100]	99.3 [98.6–99.7]	KN95 (nonmedical device GB2626-2006)
Surgical 1	99.9 [99.8–100]	99.5 [98.7–99.5}	Level 1 single use surgical mask (according to AS 4381:2015 Nelson Laboratories, USA, bacterial filtration efficacy (BF) average 98.2%, minimum 97.1% as per ASTMF1862)
Surgical 2	99.6 [99.3–99.8]	98.5 [98.3–98.6]	Surgical face mask (99.9% BFE ^2^)
Disposable 1	99.9 [99.9–100]	99.7 [99.7–99.9]	Disposable face mask (nonmedical GB/T32610-2016)
Fabric 1	54.4 [54.3–54.6]	65.8 [64.1–67.6]	Three layered masks made of 100% cotton
Fabric 2	67.3 [54.8–92.1]	90.9 [86.5–94.3]	Denim face mask—double layer stretchy cotton
Fabric 3	93.6 [92.1–96.3]	89.0 [86.1–90.5]	100% hemp outer layer, poly membrane mid layer, and organic cheesecloth inner layer
Fabric 4	50.3 [49.7–51.2]	63.6 [51.8–75.0]	Two layers of 100% Mulberry Silk
Fabric 5	54.9 [55.4–55.7]	93.32 [86.9–97.7]	Washable fabric face mask with pocket for filter made from cotton and poplin fabric
Fabric 5 + dried baby wipe	98.5 [97.7–99.6]	97.6 [97.0–98.5]	Fabric 5 with a dried baby wipe inserted into the pocket
Fabric 5 + vacuum cleaner bag	99.5 [98.9–99.9]	98.8 [96.9–99.8]	Fabric 5 with a section of a vacuum cleaner bag inserted into the pocket
Fabric 6	98.6 [97.7–99.6]	99.1 [98.3–99.7]	Made using the Victorian DHHS design [13]. Two layers of reusable shopping bag (nonwoven polypropylene) and one layer of cotton

All masks were tested in triplicate except Fabric 1, which was tested in duplicate. The average aerosol size that the masks were tested against was 6.0 µm and the viral filtration efficiency was calculated using this aerosol size and then again with the larger aerosol excluded to give an average aerosol size of 2.6 µm to better represent the size of aerosols that reach the lower respiratory system. ^1^ Description information was collected from the mask packaging or seller website. ^2^ Bacterial filtration efficiency.
